# Noise Exposure of Teachers in Nursery Schools—Evaluation of Measures for Noise Reduction When Dropping DUPLO Toy Bricks into Storage Cases by Sound Analyses

**DOI:** 10.3390/ijerph13070677

**Published:** 2016-07-04

**Authors:** Konstanze Gebauer, Thomas Scharf, Uwe Baumann, David A. Groneberg, Matthias Bundschuh

**Affiliations:** 1 Institute of Occupational Medicine, Social Medicine and Environmental Medicine, Goethe-University of Frankfurt, Theodor-Stern-Kai 7, 60590 Frankfurt am Main, Germany; konstanze.gebauer@kgu.de (K.G.); tcscharf@googlemail.com (T.S.); groneberg@med.uni-frankfurt.de (D.A.G.); 2 Department of Otorhinolaryngology, Goethe-University of Frankfurt, Theodor-Stern-Kai 7, 60590 Frankfurt am Main, Germany; uwe.baumann@kgu.de

**Keywords:** nursery schools, noise intervention measures, sound analyses, occupational health

## Abstract

Background: Although noise is one of the leading work-related health risk factors for teachers, many nursery schools lack sufficient noise reduction measures. Methods: This intervention study evaluated the noise exposure of nursery school teachers when dropping DUPLO toy bricks into storage cases. Sound analyses of the impact included assessment of the maximum sound pressure level (L_AFmax_) as well as frequency analyses with 1/3 octave band filter. For the purpose of standardization, a customized gadget was developed. Recordings were performed in 11 cases of different materials and designs to assess the impact on sound level reduction. Thereby, the acoustic effects of three damping materials (foam rubber, carpet, and PU-foam) were investigated. Results: The lowest L_AFmax_ was measured in cases consisting of “metal grid” (90.71 dB) or of a woven willow “basket” (91.61 dB), whereas a case of “aluminium” (103.34 dB) generated the highest impact L_AFmax_. The frequency analyses determined especially low L_AFmax_ in the frequency bands between 80 and 2500 Hz in cases designs “metal grid” and “basket”. The insertion of PU-foam achieved the most significant attenuation of L_AFmax_ (−13.88 dB) and, in the frequency analyses, the best sound damping. Conclusion: The dropping of DUPLO bricks in cases contributes to the high noise level in nursery schools, but measured L_AFmax_ show no evidence for the danger of acute hearing loss. However, continuous exposure may lead to functional impairment of the hair cells and trigger stress reactions. We recommend noise reduction by utilizing cases of woven “basket” with an insert of PU-foam.

## 1. Introduction

Nursery schools are among the fields of work, which record the largest increase of noise-induced effects on health [1]. Noise-induced hearing impairment is the only recognized noise related occupational disease [2,3,4] and represents 42.3% of the most common in Germany [5,6]. Furthermore, noise is one of the strongest work-related stress factors for teachers in nursery schools [6,7]. Based on occupational studies, noise was subjectively assessed as distressing by the teachers [8,9] and up to 82% of the teachers reported to feel burdened by noise [10]. Interviews with teachers concerning negative working conditions and stress factors showed that noise (73%) is the third strongest occupational risk [10]. Some objective noise measurements in nursery schools showed maximum equivalent sound pressure levels over 8 h (L_eq8h_) of up to 90 dB (A) in particular for the supervision of children in playing sections [11,12,13]. The average L_eq8h_ in nursery schools is in the range of medium intensity of 60–85 dB [1,8,9,14,15], which can typically trigger extra-auditory effects. Often-complained adverse health effects are increasing psychological strain of noise-induced stress, vocal load, masking, and higher frequency of errors [8,16].

Noise annoyance is a common effect of noise with high sound levels. Eysel-Gosepath et al. reported that annoyance in preschool is dependent on several additional parameters. The stress level is, for example, less for older teachers and full-time employees, which indicates that there is a subjective rating [17]. The study of Sjodin et al. pointed out that there is no significant correlation between the subjective noise annoyance and the objective noise measurement [8]. The level of annoyance is not only influenced by the sound pressure level but also by physical characteristics, such as the frequency spectrum, the duration of exposure, or tonal and impulse components [18]. Sounds with low frequencies are reported to cause stronger annoyance reactions, especially when including impulse tones or vibrations [19].

However, not only a high L_eq8h_ throughout a working day is critical for health, but also short sound pressure level peaks contribute to the noise hazard. Despite the fact that most average L_eq8h_ are under the threshold of hearing impairment, teachers often complain of tinnitus or acute hearing loss [15,20]. The peak values of individual measurements in nursery schools were up to 113–117 dB (A)[17] and occurred particularly in free playing sections [17,21]. Peak values of more than 130 dB (A) may cause an acute acoustic trauma. Sound events with levels of higher loudness and sudden occurrence represent stress factors in particular, as they may lead to startle responses, distraction, and annoyance [22]. Such unpredictable and uncontrollable impulse noise events present a greater burden since there is no opportunity for the individual to prepare for the impact. They further involve the hazard that the attention of the current activity is directed to the noise source [23,24,25]. This deflection, in turn, increases the risk potential and elevates frequency errors, which is particularly dangerous in child care.

One of these intense noise events in nursery schools is the situation of dropping DUPLO toy bricks (LEGO, Billund, Denmark) into storage cases at the end of a playing section. The impacts of the bricks on the bottom of the case do generate very intense noise emissions. The aim of the current study is the investigation of measures for effective noise reduction during this action, which can be achieved with minimal effort. It is hypothesized that the design of storage cases is responsible for the intensity of the impact noise and depends on the applied material. Furthermore, specific analyses should investigate whether the intensity of the dropping noise can potentially induce hearing loss and whether a specific modification of a case by incorporating different damping materials can significantly reduce noise emission.

## 2. Method

### 2.1. Material

**Figure 1:**
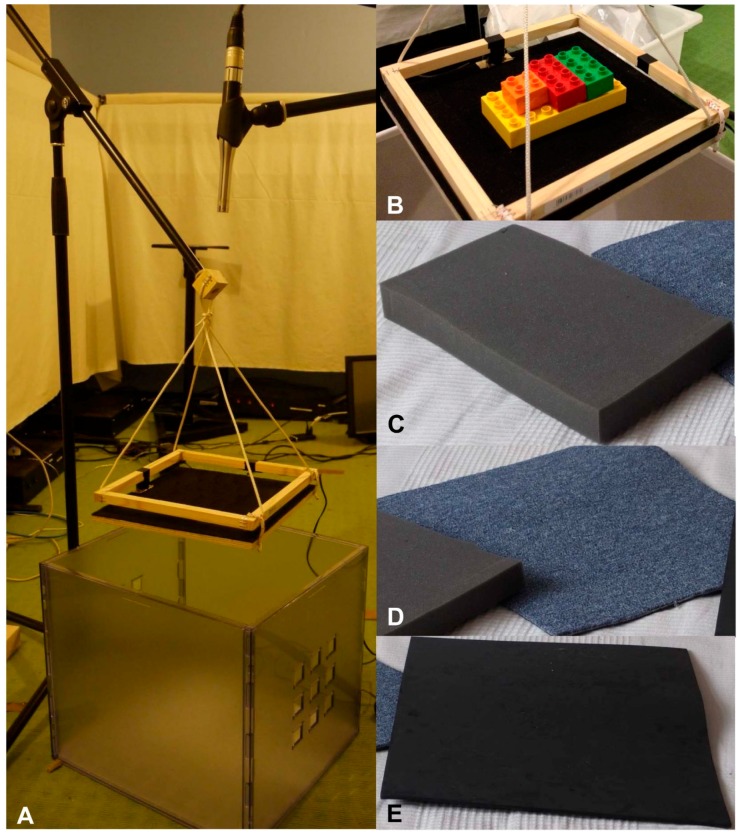
Experimental setup and damping materials: (**A**) self-constructed setup; (**B**) arrangement of selected DUPLO bricks; (**C**) PU foam; (**D**) carpet; and (**E**) foam rubber.

The noise measurements were performed with DUPLO toy bricks of the box LEGO DUPLO 6176—the basic building blocks for children aged 1½ to 5 years. For recording reproducible results the same number and kind of bricks were used for each test execution. Five different bricks that fit into two hands were selected. One 4 × 8 brick, two 2 × 4 bricks, and one 2 × 2 brick were chosen (Figure 1B).

**Figure 2:**
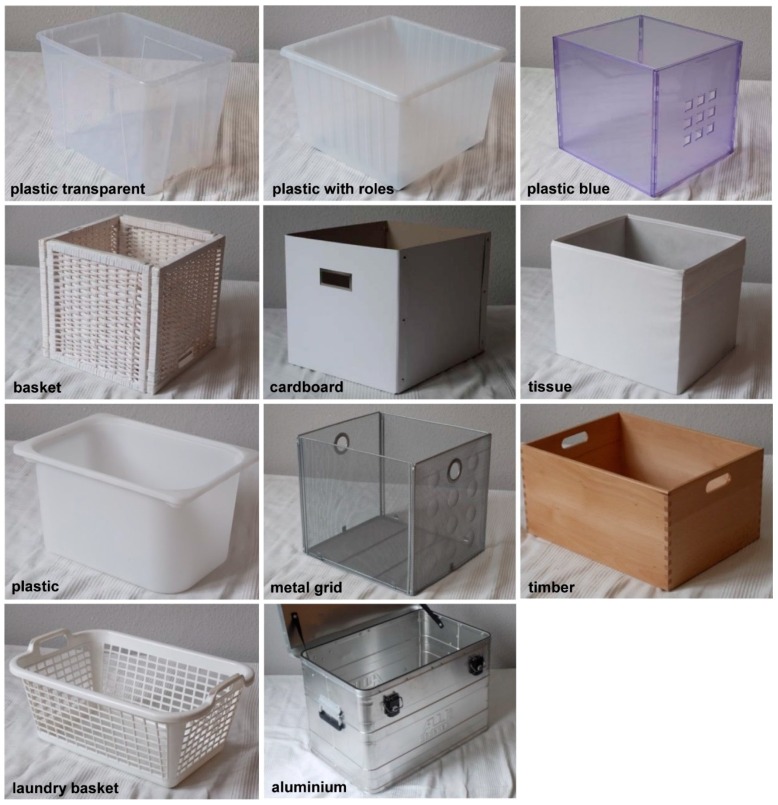
Storage cases of different materials and designs investigated by sound analyses.

The material of the selected storage cases were aluminium, willow (basket), steel (metal grid), polypropylen (plastic blue, plastic transparent, plastic with rolls, plastic white, laundry basket), beech (timber), cardboard and cardboard covered by tissue (tissue) (Figure 2). Three different damping materials were chosen to assess noise reduction and fixed to the bottom and the walls of the cases. The foam is composed of polyurethane (PU) with a height of 3 cm (Figure 1C). The height of carpet (Figure 1D) is approximately 1 cm and foam rubber, which consisted of ethylenvinylacetate, of a thickness of 0.4 cm (Figure 1E).

The sound pressure level meter XL2 (NTi Audio, Schaan, Liechtenstein) with the integrated microphone M2210 to standard DIN EN 61672 was used for measurement. The microphone was positioned by means of standardization at a height of 82 cm to represent the ear height of a woman in a heel seat. According to ISO 1996-1, for analysing single sound events the maximum sound pressure level (L_AFmax_) was used.

### 2.2. Principle of Test

For each storage case 25 measurements of the L_AFmax_ were performed under equivalent conditions. For the evaluation of the damping materials the inlays were implemented into the three cases with the highest L_AFmax_. To assess the most effective combination for noise reduction the case with the second-lowest L_AFmax_ (due to lower standard deviation) was tested with the most effective damping material. For the purpose of frequency analyses, five additional measurements of each case and combination were recorded.

### 2.3. Setting

This experimental setup is designed to provide statistical accuracy. A method to mimic the operation of dropping DUPLO toy bricks into storage cases with the highest reproducibility was developed. The measurements were performed in an anechoic chamber. The experiment was designed to simulate a typical situation in nursery schools. It was assumed that a teacher kneels in a heel seat and drops toy bricks into a storage case with two hands. A defined drop height was realized by a gadget created of a wooden trapdoor surrounded by a frame. At one side the frame was loosely attached by ropes, at the opposite side an electric magnet was arranged. By deactivating the magnet, the wood panel hinges down. For adjusting the height, the frame was fixed with ropes to a tripod. The selected height was based on the assumption that a teacher is filling the storage case just above the cases upper edge. For standardization a fixed value of 41 cm was chosen (Figure 1A). Initially, the toy bricks were positioned in a specific order at a defined position (Figure 1B). A person outside the room disables the magnet via a remote control.

### 2.4. Statistical Methods

All statistical analyses and graphics of the L_AFmax_ measurement were done by GraphPad Prism^®^ Version 6.0 (GraphPad Software, San Diego, CA, USA). To evaluate the average L_AFmax_, the mean values and the standard deviation (SD) were calculated. The tests for normal distribution were done by using a histogram and two statistical tests, the Shapiro Wilk test and D’Agostino-Pearson K2 omnibus test. The results showed a normal distribution. For analysing statistical significance, one-way ANOVA was used. The independent variable was chosen to be L_AFmax_. Afterwards, the Tukey post hoc test with a significance level of 5% was conducted, which analyses group differences between paired means by pairwise multiple comparisons. The *p*-value were chosen as non-significant: *p*-value > 0.05, significant: *p*-value ≤ 0.05, high significant: *p*-value < 0.01, highly significant: *p*-value < 0.001. To determine the best damping material, a paired sample *t*-test was applied. By means of the F-test, the normal distributed results were tested for homogeneity of variance. Since there was no equality of variance the comparison of the mean values were performed by a Welch corrected t-test with a significance level of 5% [26].

### 2.5. Frequency Analysis

To cover the entire frequency range of the human hearing, the frequency analysis was performed in a range of 25 Hz–16,000 Hz. The sound was dispersed by a noise 1/3 octave filter and evaluated afterwards.

Normalization: By means of an audio processing tool (WavePad Audio Editor, Greenwood Village, CO, USA) the audio signals were normalised so as the largest peak amplitude of each signal was equal to the floating point value of one. Next, for comparison purposes, the L_AFmax_ of each signal was then used to scale the normalized, digitized audio signal.

The frequency analyses of the normalised and scaled signals were performed and graphed by program MATLAB (Version R2011a, 7.1 MathWorks, Ismaning, Germany). The average 1/3 octave band frequency spectrum of three measurements for each noise signal case condition was calculated by a program written with MATLAB.

## 3. Results

### 3.1. Analyses of Sound Pressure Level

The L_AFmax_ analyses of the 11 different storage cases showed a range between 90.71 dB and 103.34 dB.

The highest L_AFmax_ were radiated by cases made of “aluminium” with 103.34 dB and “timber” with 101.17 dB. The lowest L_AFmax_ were measured in cases made of “basket” and “metal grid” with L_AFmax_ of 91.61 dB and 90.71 dB (Table 1).

**Figure 3:**
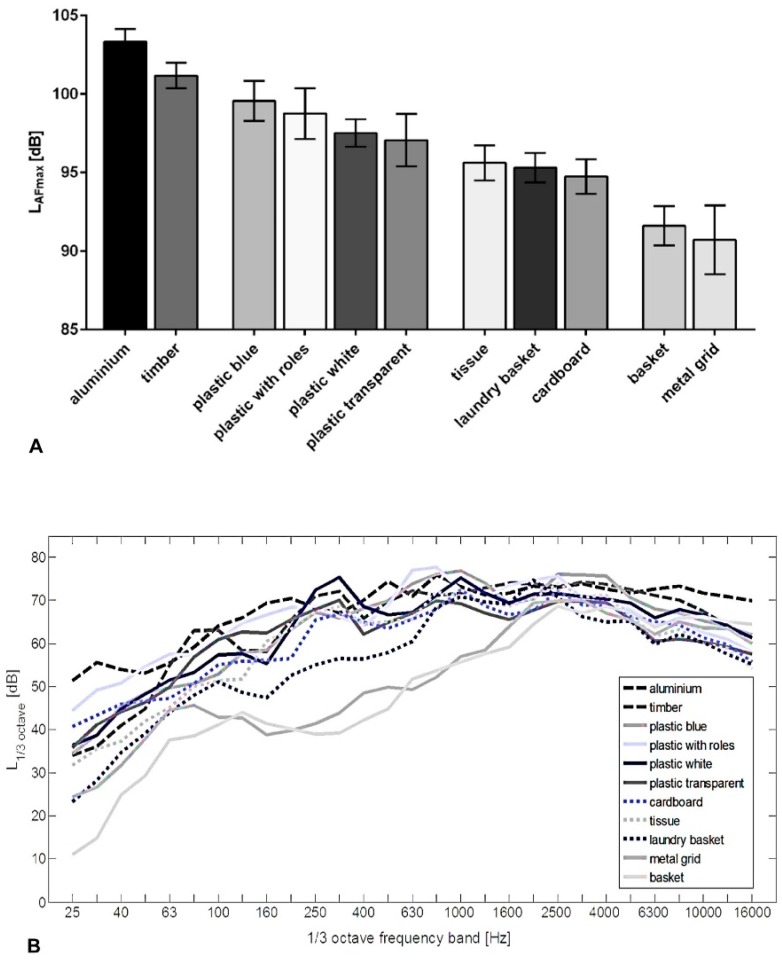
(**A**) LEGO Duplo toy brick dropping noise level measurements (L_AFmax_) of different storage cases (*n* = 11), arranged in descending order and in groups of similar L_AFmax_; and (**B**) band pass (1/3 octave band width) frequency analyses of the noise level measurements.

For further analyses, results were arranged in descending order, as well as summarized in four averaged noise level classes (Figure 3A). In the first class, a L_AFmax_ over 100 dB was reached. The next group contained the four cases made of plastic with similar noise levels (L_AFmax_ difference in between 2.49 dB). With 99.55 dB the case “plastic blue” showed the highest value in this group. In descending order, the cases “plastic with wheels”, “plastic white”, and slightly below “plastic transparent” follow. Class 3 was composed of the case “tissue”, the “laundry basket” case, and “cardboard” case (range 0.88 dB).

The ANOVA analyses showed that only six comparisons showed no significant results, which concerned, particularly, the arrangements within classes 2–4 (Table 1). The most significant differences were present by comparing the two cases with highest noise level, “aluminium” and “timber”, with the lowest noise level cases, “basket” and “metal grid” (reduction of the L_AFmax_ between 9.56 dB and 12.64 dB).

### 3.2. Sound Pressure Analyses of the Damping Materials

The capability of damping materials to reduce noise depending on the design of the storage case was analysed (Table 2). Hereby the most distinct attenuation was shown by the insertion of PU foam (−13.88 dB (A) case “timber”) and carpet (−12.00 dB (A) case “aluminium”).

**Figure 4:**
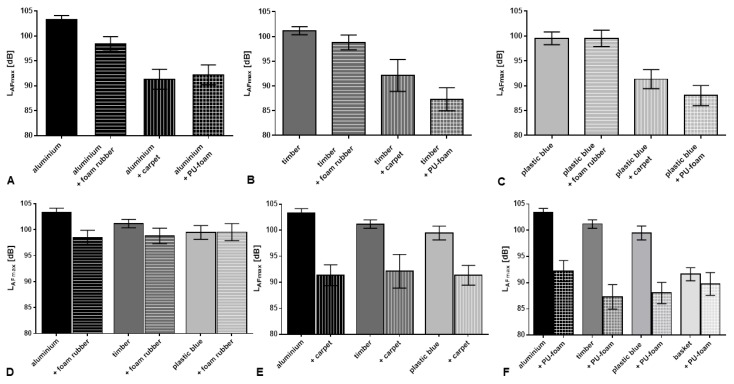
LEGO Duplo toy brick dropping noise level (L_AFmax_) measured in different storage cases. (**A**) “aluminium”; (**B**) “timber”; and (**C**) “plastic blue”, with different insert damping materials; lower row: as (**A**)–(**C**), but arranged to compare the efficacy of different insert damping materials (**D**) foam rubber; (**E**) carpet; and (**F**) PU foam.

The L_AFmax_ measurements obtained with the case “aluminium” compared to the attenuated combination showed that foam rubber achieved the smallest noise level reduction (4.90 dB). A distinct higher damping was present by applying inlays of carpet or PU foam, whereby the reduction obtained by carpet inlays was most effective. These damping materials decreased the sound pressure level by an average of 11 dB–12 dB, respectively (Figure 4A).

The analyses of the damping effects of the three materials applied to the case “timber” showed likewise varying attenuation effects. Foam rubber inlays reached only a slight L_AFmax_ reduction of 2.37 dB, whereas inlays of carpet could achieve a distinct reduction of 9.06 dB. The strongest damping effect (13.88 dB) was observed by insertion of the PU foam inlay. The difference between these damping materials was highly significant (*p* < 0.001) (Figure 4B).

The investigation of inlay related damping effects with the case “plastic blue” identified a very slight reduction of 0.03 dB by foam rubber material which was not significant. For the insert of carpet, a strong attenuation of 8.22 dB could be determined. An even higher reduction of 11.52 dB was related to the application of PU -foam inserts. The analyses of the impact of carpet and PU-foam on noise level reduction acquired highly significant results compared to the undamped case and to the application of foam rubber inserts (*p* < 0.001). In addition, noise level reduction obtained by application of PU foam is highly significantly stronger compared to carpet insert (Figure 4C). 

The analysis of inlay damping effects confirmed for the case “basket” with insert of PU foam a highly significant reduction (1.86 dB) compared to the reference measurement.

### 3.3. Efficacy of the Damping Materials

The damping effect of the different inserts was depending on the case type/material. Foam rubber reached different grades of damping. Applied to the case “plastic blue”, no reduction of L_AFmax_ was achieved, whereas applications to “timber” (−2.37 dB) and “aluminium” (−4.90 dB) cases revealed more effective attenuation (Figure 4D).

The analyses of the ameliorating impact of inlays consisting of carpet material evaluated a distinct attenuation in all cases. This was most pronounced in the case made of “aluminium” (damping effect 12.00 dB). Applied to cases “timber” and “plastic blue” a smaller damping effect was present (9.06 dB and 8.22 dB) (Figure 4E).

The inlay consisting of PU foam was added additionally to the storage case “basket” (the second-lowest storage case; for testing the most effective combination). It showed the strongest damping effect (13.88 dB) applied to the case “timber”. Noise reduction was slightly smaller with application to cases “aluminium” and “plastic blue” (11.13 dB and 11.52 dB, respectively). The smallest damping effect was observed with the application of PU-foam to the case “basket” (1.91 dB). This small damping effect is related to the comparably soft noise level of the basket already shown without damping material (Figure 4F).

### 3.4. Frequency Analysis

The comparison of the 1/3 octave filter output level showed the most pronounced differences between all tested case conditions in the low and mid frequencies below 2000 Hz (Figure 3B). In the mid frequencies between 250 Hz and 4000 Hz the highest sound pressure levels were present.

In the range between 80 Hz and 2500 Hz the differences in terms of filter output level in case conditions “basket”, “metal grid”, and “laundry basket” were remarkable. Whereas the sound pressure level of all other cases tend to be increased towards the lower end of the frequency range. The spectrum of cases “basket” and “metal grid” initially showed a strong attenuation. The sound pressure levels measured in these two cases were up to 30 dB weaker compared to the 1/3 octave band filtered levels of the eight other cases. To a lesser extent (−10 dB) this did also apply to the case “laundry basket”.

### 3.5. Frequency Analysis: Efficacy of the Damping Materials

Over the entire frequency spectrum of all three tested cases the insertion of foam rubber accomplished only a slight reduction of the band filtered amplitude. The distinct attenuation was achieved by insertion of PU foam and carpet inlay.

**Figure 5:**
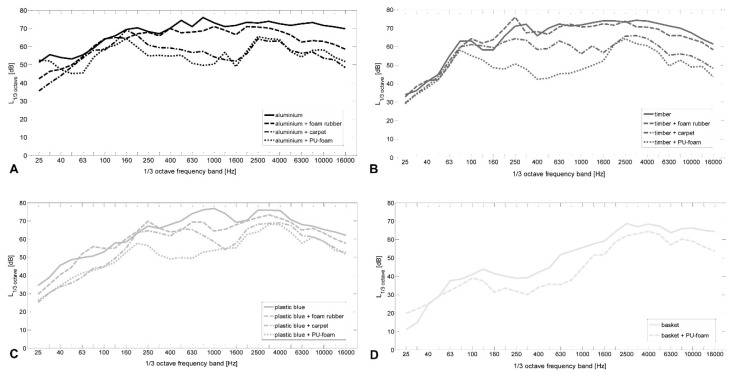
Band-filtered LEGO Duplo toy brick dropping noise spectra measured in different storage cases. (**A**) “aluminium”; (**B**) ”timber”; (**C**) “plastic blue”; and (**D**) “basket”. Impact of different insert damping materials (foam rubber, carpet, PU foam).

The frequency-specific analyses of the noise generated with case condition “aluminium” indicated that all tested damping materials caused a reduction of the sound pressure amplitudes predominantly in the mid and high frequencies (Figure 5A). Across the whole spectrum a stronger damping effect was achieved by the insertion of carpet and PU foam inlays, especially up to 2500 Hz. In the range above 2500 Hz the damping effect was smaller for all materials.

For case condition “timber” the most distinct damping effect covering the complete spectrum was achieved by insertion of PU foam. The damping effect was most pronounced in the mid frequencies between 160 Hz and 2500 Hz. Up to 80 Hz and above 2500 Hz the attenuation obtained with carpet and PU foam inlays differed only slightly and the spectra were nearly identical (Figure 5B).

In case condition “plastic blue” the highest damping effect was observed covering the whole spectrum by insertion of PU foam inlays. This damping effect was particularly pronounced in the mid frequencies between 200 Hz and 1000 Hz. Within the low frequencies up to 160 Hz, as well as in the high frequencies above 2500 Hz, the damping effect obtained with carpet and PU foam inlays was slightly varying but showed a similar trend (Figure 5C).

In case condition “basket” the insert of PU foam enabled a frequency independent attenuation on an approximately 10 dB lower level compared to the other case conditions (Figure 5D). The comparison of the damping effect of PU foam inserted into case “basket” with “aluminium”, “timber”, and “blue plastic” revealed a less pronounced attenuation in the mid frequencies.

## 4. Discussion

When inserting Duplo bricks into storage cases many different physical effects have an influence on the L_AFmax_. The insertion process itself, the dropping height of the blocks, or the material properties of the Duplo are examples of important factors. For standardization, a height of 41 cm was chosen to avoid an alteration of the kinetic energy of the bricks. However, one of the most dominant effects on the L_AFmax_ is the box design and its acoustic properties. It is the focus of this publication to analyse the influence of common box designs on the noise annoyance of nursery school teachers and how common shock-absorbing materials can contribute to a L_AFmax_ reduction. To delimit the effect of the box design from other factors a standardized method for the insertion process with a fixed dropping height of the Duplo bricks was developed.

The results of the L_AFmax_ measurements revealed that the process of dropping DUPLO toy bricks into storage cases contribute to the high noise levels in nursery schools. However, the noise level determined in the present study were not in the range of the observed maximum values of up to 117 dB (A) [17]. The analyses showed noise levels in the range of 90.71 dB for the storage case “metal grid” to 103.34 dB for the case condition “aluminium”. The extent of the noise emission depends on the material and construction of the storage case. The material properties are crucial for the intensity of the structure-borne noise or airborne noise of the interacting parts. The internal damping of materials describes the heat loss which arises due to friction at the atomic and molecular level under mechanical stress. This depends on the dissipation factor η [27]. For plastics, the dissipation factor of η = 10^−1^ is markedly higher compared to wood and aluminium, with η = 10^−2^ or η = 7 × 10^−5^. The storage cases “laundry basket”, “basket”, and “metal grid” present perforated walls and differ structurally from the other cases. As a consequence, the spreading of the structure-borne sound is impeded and less material exists for airborne sound stimulation. This may explain that the cases made of steel “metal grid” has the lowest noise emission despite the low dissipation factor of η = 10^−4^.

Cases made of plastic generated equivalent sound pressure levels so that the effects of slight design differences are small compared to the impact related to the construction material. Furthermore, the amount of noise emission observed in cases made of plastic differs highly or is highly significant from those cases made of materials such as timber, aluminium, basket, tissue, and cardboard.

Several studies on noise reduction measures in nursery schools investigated the impact of more quiet toys and damped tables, and demonstrated a remarkable noise damping effect as reflected by L_Eq8h_ reduction up to 10 dB (A) [1,28]. To our knowledge, only one single study in a nursery school measured the L_AFmax_ of dropping DUPLO toy bricks out of a case on a stone floor where a L_AFmax_ of 109 dB (A) and on a wooden floor a L_AFmax_ of 102 dB (A) was reported [17]. Compared to the damping effect of 7 dB related to the floor construction, the replacement of the case “aluminium” by that of a basket or metal grid shows a higher damping effect of up to 12.64 dB.

Concerning the potential harmful effects of the L_AFmax_ noise levels measured in the present study, the impulse noise generated by dropping DUPLO toy bricks into different cases was within the range where energetic damage of the inner hair cell cilia can occur due to metabolic overload independent from the type of material and construction of the case. Furthermore, the exposure of sound pressure levels of 100 dB (A) for 10–20 min elevates the risk of disturbance of the microcirculation in the inner ear [29,30,31]. Following the usage of cases of class 1, “aluminium” and “timber” elevates the risk of hearing impairment in an equivalent exposure time. As a consequence the replacement of a storage case from class 1 by one of class 4 has a positive effect on prevention and may reduce the risk of possible hazards. Although the reported L_AFmax_ in nursery schools is as high as 117 dB, this noise level is not within the damaging range of exposures for acute hearing impairment. However, almost 50% of teachers complain of hearing deficiency and tinnitus[14,15]. Thus, it could be assumed that a risk of hearing impairment is also due to permanent exposure to short sound events, even if the exposure action level of 137 dB (C) for impulse noise is not exceeded. Therefore, dropping of DUPLO toy bricks into cases could potentially contribute to the noise-related inner ear hazard.

In addition to the sound pressure level and the duration of exposure, the spectral composition of sound events is also potentially related to damage of the auditory system. With noise consisting of high energy in the higher frequencies, the risk for temporary threshold shift is elevating. High noise levels with predominant frequencies at around 4000 Hz is associated with aural impairment [18]. In the further progression the noise-related impairment spreads first to the high and then to the mid frequencies between 500 Hz and 2000 Hz [32]. In the range between 80 Hz and 2000 Hz, the case conditions “metal grid”, “basket”, and especially the case “laundry basket”, showed the lowest sound pressure level and, therefore, have a favourable effect on preventing the hair cell cilia in this frequency range from damage. However, in all cases, maximum noise levels were present between 2000 Hz and 4000 Hz, the maximum of the sensitivity range, indicating a possible interference in this susceptible area.

The most common extra-aural effect of noise is annoyance [16]. Studies which compared the degree of high sound pressure to the level of annoyance showed that there is no distinct relation between the reduction of noise and decreased annoyance. As a consequence there are other factors like pulse characteristics which are contributing to the level of annoyance. The impact of DUPLO toy bricks on hard surfaces presents noise events with high peak sound pressure levels and sudden onset, which contributes to an increased risk of hearing damage [22,33]. It has been shown that a decrease of at least 4 dB is required to achieve a significant change in terms of the degree of annoyance [34]. A study of airport noise revealed that changes of less than 3 dB were not perceived by the neighbourhood [35]. Since, in this study, a significant reduction of the sound pressure level of about 12.71 dB was measured between the loudest and quietest case condition, a reduction in noise annoyance for this sound event can be assumed. As the loudest case conditions of each class differ by 4 dB L_AFmax_, already the replacement of a case by one belonging to a class of lower noise emission level allows a noticeable noise reduction. Furthermore, since the differential threshold between two sound pressure levels is 1 dB, even the noise reduction between the lowest and the second-lowest storage case might be perceivable [36]. Chronic effects caused by possible stress reactions could, therefore, be restrained. Frequency analyses evaluated that the highest amplitudes of all tested case conditions mainly occurred in the mid to high frequencies of 200 Hz–4000 Hz. This is consistent with the fact that high-frequency components are typical for an acoustic excitation due to a sudden impact with short pulse duration. The cases “laundry basket”, “metal grid”, and “basket” showed the lowest amplitudes over the whole spectrum. Most noteworthy are the low and mid frequencies from 80 Hz to 2500 Hz. While the remaining cases recorded increasing values in this range, these three cases showed decreased amplitudes. The special construction of the two cases with perforated walls (“metal grid” and “basket”) could be causative for the similar frequency characteristic. This effect is, to a lesser extent, also observable for the “laundry basket” due to the greater openings in the walls.

In nursery schools prevalent frequencies of 50 Hz–150 Hz and 500 Hz–4000 Hz were measured [11,37,38]. The presence of two prevalent frequency ranges has been attributed to the material of the device, the use of toys and the amount of children’s voices. The frequency measurements of this study showed the highest amplitudes mainly in the mid to high frequencies of 200 Hz–4000 Hz. The dropping of DUPLO toy bricks in storage cases could, therefore, contribute to the prevalent frequencies in nursery schools.

For an effective noise reduction, the acoustic impacts of three damping materials were additionally tested. The easiest and most effective way to reduce noise is the application of materials that mitigate against the impact due to its soft, malleable, and resilient structure and, therefore, reduce the induction of oscillation and noise emission. Decisive is a high dissipation factor and a low stiffness, which are present in foams and carpets. The most distinct attenuation was shown by the insertion of PU foam and carpet inlays into the cases. The damping effect differed only slightly depending on the case material.

The frequency analyses revealed for PU foam the best sound dampening characteristics over the whole spectrum for all cases. Attenuation was particularly achieved in the mid frequencies between 160 Hz and 2500 Hz. Since the main language area is located in this attenuated range this may contribute to reduce interference in the understanding of speech. Over the whole spectrum the attenuation effect of carpet was less than the attenuation of PU foam but was slightly varying between the three tested cases. For the foam rubber only a slight reduction in the entire frequency range could be analysed in all cases.

The analysis also indicates that the construction of the cases is less important when they are modified by either foam rubber or PU foam inlays. When applying the same materials the different storage cases showed nearly the same sound pressure level. This finding is of practical interest because a suitable damping material as PU foam could attenuate any storage cases and, therefore, many preschools would not need to purchase new ones.

Nevertheless, for the most effective noise reduction the case “basket” with an insert made of PU foam is recommended.

Due to the experimental design only the L_AFmax_ of the first dropping of the DUPLO toy bricks into the empty case was investigated. Even though this first drop is commonly the noisiest, this experiment only reflects a snapshot of the whole filling procedure. With an increasing number of bricks in the case, the influence of the bricks itself rises and the acoustical properties of the case do have a reduced influence. Thus, for a detailed evaluation the complete filling of the case could be measured. A further variable is whether a DUPLO brick first encounters another brick before hitting the bottom of the case; but this is rare and random. Therefore, the number of repetitions should be high in order to minimize this effect. For this, a test set-up had to be re-established. To investigate the variation related to random effects, 25 repeated measurements were carried out for each case condition. However, based on the results of the standard deviations, it has to be recognized that a SD of 2.19 dB is quite high with respect to the absolute values of the cases. To increase the validity a greater number of repeated measurements of possibly 50 is recommended. Further studies should evaluate other possibilities of noise reduction for this action; for example, the production of DUPLO bricks which generate lower sound pressure levels.

## 5. Conclusions

When dropping DUPLO toy bricks into different cases, the highest L_AFmax_ was measured for the case made of “aluminium” with 103.34 dB, and the lowest L_AFmax_ for the cases manufactured of “basket” and “metal grid”, with L_AFmax_ of 91.61 dB and 90.71 dB. Frequency analyses evaluated the highest amplitudes for all tested case conditions mainly in the mid to high frequencies of 200 Hz–4000 Hz. Concerning the potential harmful effects, the measured L_AFmax_ showed no evidence for the danger of acute hearing loss. Although L_AFmax_ produced in real-life conditions could be higher than the levels measured in this study. However, the measured impact noise sound levels are in a range where the risk of hearing impairment in an equivalent exposure time is increased, especially for the loudest storage cases “aluminium” and “timber”. As a consequence, the replacement of a storage case of class 1 by one of class 4 may reduce this risk. Frequency analyses evaluated for the cases condition “metal grid” and “basket” the lowest L_AFmax_ over the entire 1/3 octave band spectrum. Remarkably small amplitudes were present in the low and mid frequency range from 80 Hz to 2500 Hz. These effects are, on the one hand, due to the resilient material which impeded the excitation of structure-borne noise and, on the other hand, to their permeable walls, which reduce early reflections of sound pressure.

The most distinct attenuation of the L_AFmax_ was shown by the insertion of PU foam and carpet into the cases, which depended just slightly on the case design.

Over the whole spectrum the frequency analyses revealed, for PU foam, the best sound dampening. Attenuation was particularly achieved in the mid frequencies between 160 Hz and 2500 Hz. Since the main language area is located in this range, this may have a favourable impact on speech intelligibility and reduces the strain in this sensitive range of the hearing.

For the purpose of a highly-significant noise reduction when inserting DUPLO toy bricks a case, a resilient material with permeable walls is recommended due to its favorable impact on sound emission. Furthermore, for achieving an effective attenuation with minimal effort, an inlay of PU foam should be applied.

## Figures and Tables

**Table 1:**
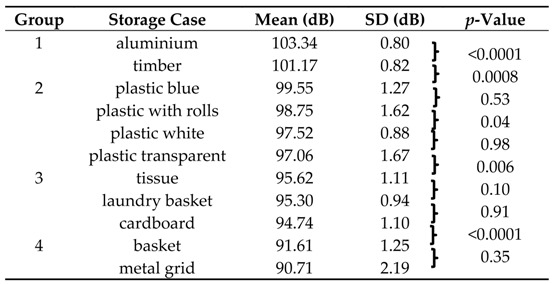
Descriptive and statistical data of L_AFmax_ noise level measurements when dropping DUPLO toy bricks. Eleven different storage cases are arranged in classes of decreasing order. Statistical significance analyses were performed with one-way ANOVA and following Tukey post hoc test. It was iteratively done for each basket with its adjacent case in the decreasing L_AFmax_ order. Calculation of SD was performed with *n* = 25 and Bessel’s correction.

**Table 2:** Acoustic effects of different damping materials applied to storage cases “aluminium”, “timber”, “plastic blue”, and “basket”. Statistical significance analyses were performed with *n* = 25 and using a paired sample *t*-test with Welch correction.

Storage Case	Mean (dB)	SD (dB)	Reduction of L_AFmax_ (dB)	*p*-Value
aluminium	103.34	0.80		
+ damping material:				
foam rubber	98.44	1.45	4.90	<0.0001
carpet	91.34	2.00	12.00	<0.0001
PU-foam	92.22	2.00	11.12	<0.0001
Timber	101.17	0.82		
+ damping material:				
foam rubber	98.80	1.49	2.37	<0.001
carpet	92.11	3.23	9.06	<0.001
PU-foam	87.29	2.33	13.88	<0.001
plastic blue	99.55	1.27		
+ damping material:				
foam rubber	99.52	1.65	0.03	>0.999
carpet	91.33	1.91	8.22	<0.001
PU-foam	88.03	2.02	11.52	<0.001
basket	91.61	0.25		
+ damping material:				
PU-foam	89.75	0.43	1.86	0.0023

## References

[1] Sjödin F., Kjellberg A., Knutsson A., Landström U., Lindberg L. (2014). Measures against preschool noise and its adverse effects on the personnel: An intervention study. Int. Arch. Occup. Environ. Health.

[2] Ising H., Kruppa B. (2004). Health effects caused by noise: Evidence in the literature from the past 25 years. Noise Health.

[3] Moshammer H., Kundi M., Wallner P., Herbst A., Feuerstein A., Hutter H.-P. (2015). Early prognosis of noise-induced hearing loss. Occup. Environ. Med..

[4] Hong O., Kerr M.J., Poling G.L., Dhar S. (2013). Understanding and preventing noise-induced hearing loss. Disease-a-Month.

[5] Rothe I., Arbeitsmedizin B.F.A.U. (2014). Sicherheit und Gesundheit bei der Arbeit 2013—Unfallverhütungsbericht Arbeit.

[6] Losch D., Schulze J. (2015). Stressfaktoren in kindertagesstätten. Zent. Arbeitsmed..

[7] Eysel-Gosepath K., Daut T., Pinger A., Lehmacher W., Erren T. (2012). Sound levels and their effects on children in a german primary school. Eur. Arch. Oto-Rhino-Laryngol..

[8] Sjödin F., Kjellberg A., Knutsson A., Kjellberg A., Landström U. (2012). Noise and stress effects on preschool personnel. Noise Health.

[9] Eysel-Gosepath K., Daut T., Pinger A., Lehmacher W., Erren T. (2012). Effects of noise in primary schools on health facets in german teachers. Noise Health.

[10] Khan A., Thinschmidt M., Seibt R. (2006). Workplace health promotion for nursery school teachers. Präv. Gesundh..

[11] Perego L., Bertoni G., Goglio F., Giovannelli G. (1996). Children and noise. Eur. J. Epidemiol..

[12] Grebennikov L., Wiggins M. (2006). Psychological effects of classroom noise on early childhood teachers. Aust. Educ. Res..

[13] Kemp A., Delecrode C., Guida H., Ribeiro A., Cardoso A. (2013). Sound pressure level in a municipal preschool. Int. Arch. Otorhinolaryngol..

[14] Seibt R., Dutschke D., Thinschmidt M., Khan A. (2004). Netzwerk für gesunde beschäftigte in kindertagesstätten—Projektkonzept, umsetzung und erste befunde. Z. Arb..

[15] Sjödin F., Kjellberg A., Knutsson A., Landström U., Lindberg L. (2012). Noise exposure and auditory effects on preschool personnel. Noise Health.

[16] Basner M., Babisch W., Davis A., Brink M., Clark C., Janssen S., Stansfeld S. (2014). Auditory and non-auditory effects of noise on health. Lancet.

[17] Eysel-Gosepath K., Pape H., Erren T., Thinschmidt M., Lehmacher W., Piekarski C. (2010). Sound levels in nursery schools. HNO.

[18] Mahendra Prashanth K., Venugopalachar S. (2011). The possible influence of noise frequency components on the health of exposed industrial workers—A review. Noise Health.

[19] Leventhall H. (2004). Low frequency noise and annoyance. Noise Health.

[20] Martins R.H., Tavares E.L., Lima Neto A.C., Fioravanti M.P. (2007). Occupational hearing loss in teachers: A probable diagnosis. Rev. Bras. Otorrinolaringol..

[21] Truchon-Gagnon C., Hetu R. (1988). Noise in day-care centers for children. Noise Control Eng. J..

[22] Davies H., Kamp I. (2012). Noise and cardiovascular disease: A review of the literature 2008–2011. Noise Health.

[23] Landström U., Akerlund E., Kjellberg A., Tesarz M. (1995). Exposure levels, tonal components, and noise annoyance in working environments. Environ. Int..

[24] Guski R. (1999). Personal and social variables as co-determinants of noise annoyance. Noise Health.

[25] Kjellberg A., Landström U., Tesarz M., Söderberg L., Akerlund E. (1996). The effects of nonphysical noise characteristics, ongoing task and noise sensitivity on annoyance and distraction due to noise at work. J. Environ. Psychol..

[26] Motulsky H. (2007). Prism 5 Statistics Guide.

[27] Kollmann F., Schösser T., Angert R. (2006). Praktische Maschinenakustik.

[28] Södersten M., Granqvist S., Hammarberg B., Szabo A. (2002). Vocal behavior and vocal loading factors for preschool teachers at work studied with binaural dat recordings. J. Voice.

[29] Ising H., Sust C., Plath P. (1996). Lärmbeurteilung—Gehörschäden. Arbeitswissenschaftliche Erkenntnisse. Forschungsergebnisse für die Praxis.

[30] Lamm K., Arnold W. (1996). Noise-induced cochlear hypoxia is intensity dependent, correlates with hearing loss and precedes reduction of cochlear blood flow. Audiol. Neurotol..

[31] Quirk W.S., Seidman M.D. (1995). Cochlear vascular changes in respone to loud noise. Otol. Neurotol..

[32] McBride D.I., Williams S. (2001). Audiometric notch as a sign of noise induced hearing loss. Occup. Environ. Med..

[33] Casali J., Robinson G., Marras W.K.W. (1999). Noise in industry: Auditory effects, measurement, regulations and management. The Occupational Ergonomics Handbook.

[34] Laszlo H., McRobie E., Stansfeld S., Hansell A.L. (2012). Annoyance and other reaction measures to changes in noise exposure—A review. Sci. Total Environ..

[35] Fidell S., Silvati L., Pearsons K. (1998). Noticeability of a decrease in aircraft noise. Noise Control Eng. J..

[36] Möser M., Zimmermann S., Ellis R. (2013). Engineering Acoustics: An Introduction to Noise Control.

[37] Lange M. (2003). Kindertagesstätten sicher gestalten—Leitfaden für bauherren, architekten und planungsämter zur sicherheitsgerechten gestaltung von kindertagesstätten. Schriftenreihe der Unfallkasse Hessen.

[38] Verbeek J., Dijk F., Vries F. (1987). Non-auditory effects of noise in industry. Int. Arch. Occup. Environ. Health.

